# Supplementary material for: Measuring, processing, and generating partially coherent light with self-configuring optics
Source: Light Sci Appl. 2024 Sep 20;13:260. doi: 10.1038/s41377-024-01622-y (PMC11413175; doi:10.1038/s41377-024-01622-y)
Supplement: Supplementary file 1 — Supplementary Information [file 41377_2024_1622_MOESM1_ESM.pdf]

Supplementary Information for:
Measuring and processing partially coherent light with self-configuring optics

Charles Roques-Carmes[1],* Shanhui Fan[1], and David Miller[1]

[1] *E. L. Ginzton Laboratories, Stanford University,*

348 Via Pueblo, Stanford, CA 94305

*Electronic address: `chrc@stanford.edu`

Contents

S1. Partial coherent light analyzers (PCLA): architecture and learning algorithm 3

A. Background on coherency matrix 3

B. Self-configuring arrays of Mach-Zehnder interferometers (MZI) 4

C. PCLA learning algorithm 5

S2. Numerical implementation of PCLA learning algorithm 9

A. Decomposition algorithm for triangular arrays 10

B. PCLA learning algorithm in triangular arrays 11

S3. Analysis of quantum optical mixtures with PCLA 12

A. Background on density matrix 12

B. PCLA algorithm for quantum optical system 13

C. Influence of losses 15

S4. Connection to other modal representations and measurement methods of partial coherent light 15

A. Coherence tomography 15

B. Karhunen-Loève expansions 16

S5. Discussion of self-configuring algorithm for a layer with partially coherent light 17

S6. Experimental considerations 19

A. Experimental proposal 19

B. Influence of additional sources of noise on fidelity 19

References 20

S1. PARTIAL COHERENT LIGHT ANALYZERS (PCLA): ARCHITECTURE AND LEARNING ALGORITHM

In this section, we describe the architecture and learning procedure for partial coherent light analyzers (PCLA). We first focus on classical light fields described by a coherency matrix ρ.

A. Background on coherency matrix

In the following, we consider N "channels" of input light, denoted by a N-dimensional vector x. These channels can describe different spatial, polarization, or even spectral modes of light with partial coherence described by the density (also known as coherency) matrix ρ, such that $\rho_{ij} = \langle x_i x_j^* \rangle$ [1]. The matrix ρ is by definition Hermitian and semi-positive. This can be proved by considering a complex vector y:

$$\begin{aligned} y^\dagger \rho y &= \sum_{ij} y_i \rho_{ij} y_j^* \\ &= \left\langle \left| \sum_i x_i y_i \right|^2 \right\rangle \\ &\geq 0. \end{aligned}$$

As a direct consequence, one can write $\rho = UDU^\dagger$, where U is unitary and D is a diagonal of positive values $D_{ii} = \lambda_i \geq 0$.

We now describe how linear photonic transformations can modify the density matrix. We consider a linear transformation (coupling operator) M which maps a given input x to output y according to $y = Mx$. The linear transformation M also transforms the density matrix according to [2]:

$$\rho' = M \rho M^\dagger, \tag{S1}$$

where ρ' is the output density matrix. We can already see that by picking $M = U^\dagger$, one "diagonalizes" the coherency matrix, such that $\rho' = D$.

B. Self-configuring arrays of Mach-Zehnder interferometers (MZI)

PCLAs rely on self-configuring photonic networks. For concreteness, we consider self-configuring arrays of Mach-Zehnder interferometers (MZI) [3]. Several architectures exist for self-configuring MZI arrays [4], such as cascades of "diagonal lines" [3, 5], which taken together can form a triangular array [6], cascaded binary trees [5], and combinations of these [4].

Self-configuring networks have a few key properties that are essential to the design of PCLAs:

1. **Cascaded architecture.** Self-configuring networks consist of cascaded self-configuring layers. Self-configuring layers have one particular output port that is connected through the 2×2 blocks to each input port by only one path [4]. (We have called this output port the "top" output port in the main text for the sake of definiteness, though it may not physically be in that geometrical position). When illuminated by one coherent light beam over possibly all the inputs, self-configuring MZI layers can automatically "learn" settings, for instance, to route all input light to their output port [3], in a completely progressive algorithm (hence the "self-configuring" name). In self-configuring networks, the output at port k only depends on self-configuring layers 1 to k and is independent of self-configuring layers with index $j > k$.

2. **Decreasing dimensionality.** The dimensionality of the self-configuring layers in the cascade decreases with the cascade depth; the first self-configuring layer contains $N - 1$ MZIs, the second layer contains $N - 2$ MZIs, ..., and the last layer (index $N - 1$) contains 1 MZI. (This behavior is self-evident in triangular MZI arrays [6], but it applies to self-configuring networks of other architectures also [4].) The total number of degrees of freedom still allows one to implement an arbitrary unitary matrix over N degrees of freedom; the number of adjustable elements (e.g., phase shifters) in these networks is essentially equal to the number of real numbers required to specify the corresponding arbitrary complex unitary matrix.

3. **Orthogonality.** Self-configuring networks exhibit orthogonality between their self-configuring layers. Let us consider an input light field x to the self-configuring network. The network is configured to route all light from the input to the output of a given layer k. Then, if one now sends input light field $y \perp x$ to the same network (without reconfiguring its parameter weights), the output light field will be distributed over output channels $k' \neq k$ [3].

C. PCLA learning algorithm

In the case of a fully *coherent* light beam that shines on possibly all the inputs, there is a straightforward algorithm that allows us to proceed completely progressively, MZI by MZI, through a self-configuring layer, minimizing power in the "drop" port of the MZI as we adjust first ϕ and then θ in that MZI. (The "drop" port is the output from an MZI that is connected to the next layer; it is not, therefore, connected to another MZI in the same layer.) In that case, the power in the "drop" port of the MZI ends up as zero, and all the power then passes progressively as needed through the MZIs in that layer to accumulate at the "top" output of that layer. In this way, with such a single coherent input field, we can route all the power to the top output, forming a self-aligning beam coupler [5]. (By setting succeeding MZIs in the layer temporarily to the necessary cross or bar state to route the MZI power to the "top" output of the layer, we can also run this algorithm just by maximizing the output power in the "top" port [5].) This is the basic self-configuring algorithm for such a light field [3, 5]. If multiple orthogonal coherent inputs are present at once, and if each can be identified by some "tone" that the power-minimizing or maximizing detector(s) for a given layer can look for, then these beams can be automatically separated by the layers according to those identifying tones [3, 7].

In this work, though, we are trying to find the orthogonal components from the entire input field, so we do not have identifying tones. In this case, the simple "MZI-by-MZI" progressive algorithm based on power minimization in the drop port of a given MZI in a given layer [5] will not work in general. We can show this by counter example, and we have provided such a counter example below in Section S5. Though it might seem that should not now describe these as self-configuring layers, nonetheless, they still form the basis for our algorithm below. We can still define them topologically in our network [4] as layers in which one "top" output of the layer is connected by one and only one path through 2×2 (MZI) blocks to every input to the layer, and we can still usefully factorize the overall network into a succession or cascade of such layers. These layers will still self-configure, though now we use a global (multiparameter) optimization in each such layer.

Specifically, if we run a multiparameter optimization in each self-configuring layer, maximizing the output power in the "top" output port by adjusting all the phase shifters (i.e., ϕ and θ) in every MZI in the layer, as we show below, we can automatically separate the mutually incoherent parts

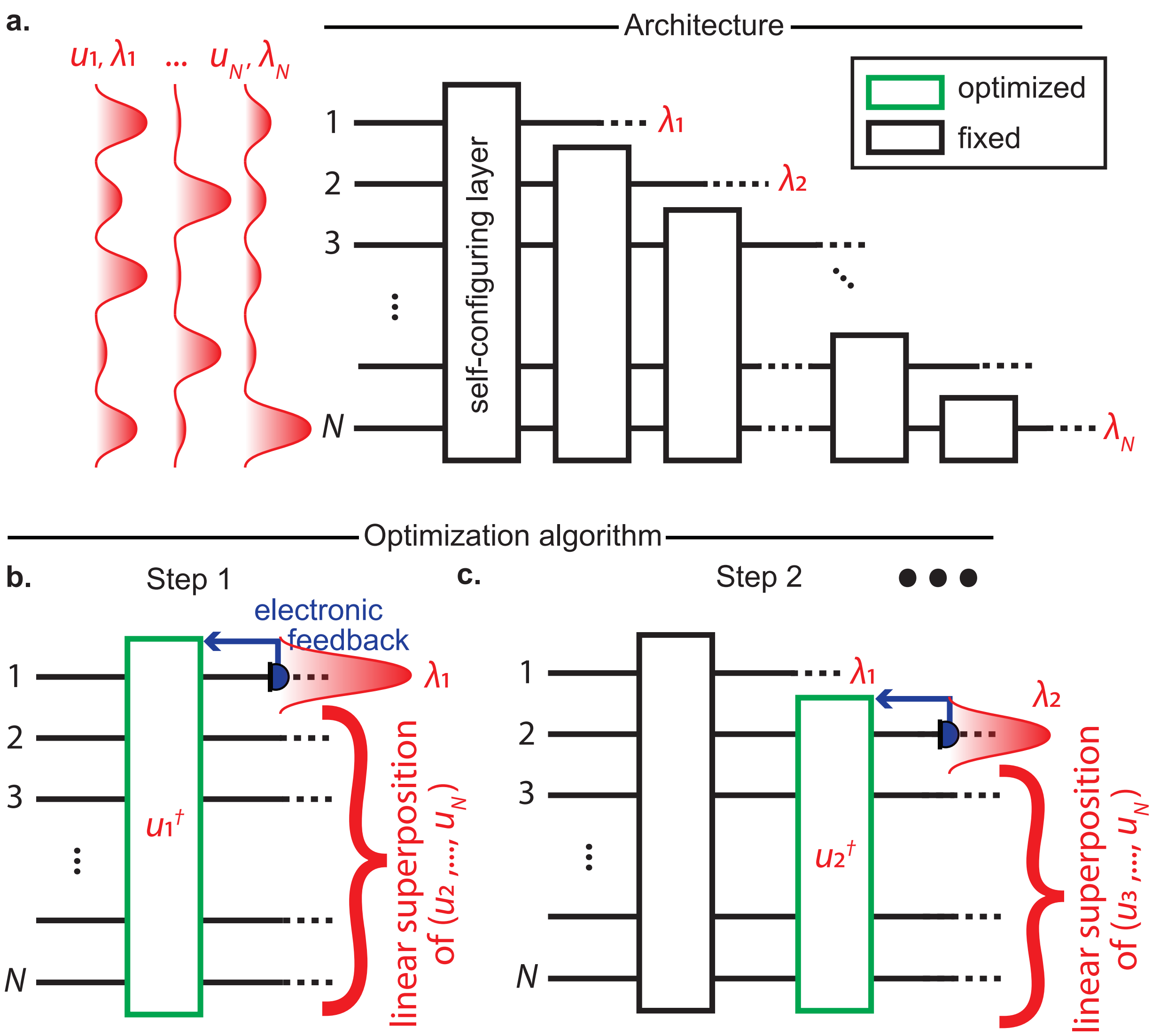

FIG. S1: **Partial coherent light analyzer (PCLA) learning algorithm. a.** In step 1 of the algorithm, the average output power of the first self-configuring layer is optimized, yielding the largest eigenvalue of the coherency matrix. **b.** In step 2 of the algorithm, the average output power of the second self-configuring layer is optimized, keeping the first layer's parameters fixed, yielding the second largest eigenvalue of the coherency matrix, and so forth. **c.** The resulting PCLA, after sequential optimization, diagonalizes the coherency matrix, and separating the input light field into its mutually incoherent parts.

of the inputs, which are the eigenfunctions of the matrix ρ. With this background, we now describe the PCLA learning method for such partially coherent fields with such self-configuring networks. We first provide a constructive proof and then a formal mathematical proof.

In the following, we consider input light characterized by coherency matrix ρ_{in} and a self-

configuring network that can be parametrized to implement a unitary transformation U_{PCLA}. For simplicity, we assume that the eigenvalues of the coherency matrix are non-degenerate (which is always the case in realistic physical settings). Degeneracy could be included in this demonstration as in the usual demonstration of the min-max theorem [8].

Before moving on to the proof, let us consider the eigenvalue decomposition of the coherency matrix:

$$\rho_{\text{in}} = UDU^{\dagger}, \tag{S2}$$

where the columns of U correspond to the set of mutually incoherent eigenmodes of the light field, and $D_{ii} = \lambda_i \geq 0$ is the average power in each such eigenmode. The eigenvalues of the coherency matrix are indexed by increasing value $\lambda_1 \geq \ldots \geq \lambda_N$. This modal decomposition of the light field allows us to find an upper bound on the power concentration into a single mode [2]. For concreteness, we rederive this bound for a given output port k. The average power in the output port is given by:

$$\langle P_k \rangle = \left(U_{\text{PCLA}} \rho_{\text{in}} U_{\text{PCLA}}^{\dagger} \right)_{kk}, \tag{S3}$$

where U_{PCLA} is the unitary transformation imparted by the self-configuring network. This can be rewritten in the form of a Rayleigh quotient:

$$\langle P_k \rangle = \langle v_k | \rho_{\text{in}} | v_k \rangle \leq \lambda_1, \tag{S4}$$

where v_k is the k-th column of $U_{\text{PCLA}}^{\dagger}$. The inequality is a known result for Rayleigh quotient and quadratic forms. This means that the maximal concentration of power is bounded by the largest eigenvalue of the coherency matrix, corresponding to the power in one of the mutually incoherent modes of the modal decomposition in Eq. S2. The optimal input field that reaches the bound is u_1, the eigenvector associated with the largest eigenvalue of ρ_{in}. This concentration bound allows us to proceed with a proof of the PCLA learning algorithm.

Constructive sketch proof. The first step of the algorithm consists in optimizing (i.e., concentrating) the output power of the first self-configuring layer. The action of the self-configuring layer is unitary. Given the eigenvalue decomposition of the partial coherent light field in Eq. S2, to maximize the average power concentrated into output port 1 (the "top" port), the self-configuring layer should map the mode corresponding to the largest eigenvalue of the coherency matrix u_1 to the first output port. This means that the first row of U_{PCLA} is determined and is equal to $u_1^{\dagger}$.

Now, let us consider the second step of the optimization. The first self-configuring layer remains fixed as per the first optimization step. Given the eigenvalue decomposition of the coherency matrix in Eq. (S2), the largest average power available on the remaining subspace corresponds to the second largest eigenvalue of the coherency matrix λ_2 with eigenvector u_2. Since the first self-configuring layer is unitary, a linear superposition of the eigenmodes $u_2, \ldots, u_n$ is sent into ports $2, \ldots, N$. In other words, we are back to the first step of the algorithm, except we have eliminated a dimension of the problem, as well as reduced the number of available waveguide ports. The average output power of the second self-configuring layer is λ_2, which corresponds to setting the second row of U_{PCLA} to $u_2^\dagger$.

Therefore, we proceed as follows with increasing step index, decreasing eigenvalue and corresponding average power to concentrate into the single "top" output port of each successive layer. By this process of successive power maximizations in the top port of each successive layer, the resulting self-configuring network has learned the eigenvectors of the coherency matrix and directly outputs its eigenvalues, corresponding to the average powers at each port. More specifically, we get $U_{\text{PCLA}} = U^\dagger$.

Formal proof. In the first step of the algorithm, we maximize the ensemble average over the first output port, which only depends on the first row M^1 of $M = U_{\text{PCLA}}$:

$$\begin{aligned} \max_M \langle P_1 \rangle &= \max_{M^1} (M \rho_{\text{in}} M^\dagger)_{11} \\ &= \max_{x \in S_1} x^\dagger \rho_{\text{in}} x \\ &= \lambda_1, \end{aligned}$$

with S_1 the unit sphere $S_1 = \{x \,; ||x||_2 = 1\}$. The last equality was obtained by using a known equality for Rayleigh quotients and quadratic forms. This bound is attained by $x = u_1$, the eigenvector associated with the largest eigenvalue λ_1 of ρ. Therefore, this first step of the optimization corresponds to a quadratic optimization program over $O(N)$ degrees of freedom. This optimization sets the first row of M to $M^1 = u_1^\dagger$. In the context of linear unitary meshes, this would correspond to constraining the optimization to the phase-shifters of the first self-configuring layer, which maps all input ports to the first output port (this optimization also entails $O(N)$ degrees of freedom).

Now, we generalize this to the k-th step of the algorithm by using the Courant-Fischer theorem (also known as the min-max or variational theorem of linear algebra). The k-th step consists in

optimizing $\langle P_k\rangle$:

$$\begin{aligned}\max_M\langle P_k\rangle &= \max_{M^k}(M\rho_{\text{in}}M^\dagger)_{kk}\\ &= \max_{x\in S_k}x^\dagger\rho_{\text{in}}x,\end{aligned}$$

where M^k is the k-th row of M. In a self-configuring network, the parameters of the self-configuring layer k map to a subspace S_k of dimension $\dim(S_k) = N-k+1$. S_k is a subspace of unitary vectors orthogonal to eigenvectors $u_1, ..., u_{k-1}$, which can also be written as $S_k = \text{Span}(u_k, ..., u_n)$ To finalize the proof, we need to show the following:

$$\max_{x\in S_k}\{x^\dagger\rho_{\text{in}}x\} = \min_S \max_x\{x^\dagger\rho_{\text{in}}x\ ;\ x\in S,\ |x|=1,\ \dim(S)=N-k+1\} = \lambda_k. \quad \text{(S5)}$$

The second equality comes from the min-max, variational, or Courant-Fischer theorem. The first equality still has to be proven to conclude this proof, which we do in the following. First, since $\dim(S_k) = N-k+1$, and elements of S_k are unitary, $\max_{x\in S_k}\{x^\dagger\rho_{\text{in}}x\} \geq \min_S \max_x\{x^\dagger\rho_{\text{in}}x\ ;\ x\in S,\ |x|=1,\ \dim(S)=N-k+1\}$. Now we consider a subspace S such that $\dim(S) = N-k+1$. S has a non-zero intersection with $\text{Span}(u_1, ..., u_k)$, so we consider x an element of their intersection. We can write $x = \Sigma_{i=1}^k\alpha_i u_i$. We also consider $y\in S_k$ and write $y = \Sigma_{i=k}^N\beta_i u_i$. We get the following inequality:

$$y^\dagger\rho_{\text{in}}y = \sum_{i=k}^{N}|\beta_i|^2\lambda_i \leq \lambda_k \leq \sum_{i=1}^{k}|\alpha_i|^2\lambda_i = x^\dagger\rho_{\text{in}}x.$$

Since this is true for all $y\in S_k$, we get $\max_{y\in S_k}\{y^\dagger\rho_{\text{in}}y\} \leq x^\dagger\rho_{\text{in}}x \leq \max_x\{x^\dagger\rho_{\text{in}}x\ ;\ x\in S,\ |x|=1,\ \dim(S)=N-k+1\}$. Since this is true for any S, we finally get: $\max_{y\in S_k}\{y^\dagger\rho_{\text{in}}y\} \leq \min_S \max_x\{x^\dagger\rho_{\text{in}}x\ ;\ x\in S,\ |x|=1,\ \dim(S)=N-k+1\}$, which proves the above equality. The result of step k of the algorithm is therefore $M^k = u_k^\dagger$.

As highlighted in this formal proof, the degrees of freedom to be tuned at each step of the algorithm have to enforce orthogonality of the rows of U_{PCLA}. This is naturally enforced in the self-configuring MZI arrays described in the previous section [3].

S2. NUMERICAL IMPLEMENTATION OF PCLA LEARNING ALGORITHM

In the following, we describe numerical simulations of the propagation of coherent and incoherent light through a MZI array and implement the optimization algorithm from the previous section.

A. Decomposition algorithm for triangular arrays

It is straightforward to calculate the required settings of self-configuring meshes to implement desired unitary transforms. The mesh settings can be calculated directly by progressively constructing the mesh that generates the columns of the adjoint matrix when we imagine running the mesh backwards [3], shining light into one "output" waveguide at a time, starting from the first layer; this approach works for any mesh composed of self-configuring layers as defined topologically in Ref. [4]. Quite generally, the corresponding matrix representing the mesh is an appropriate product of the 2×2 matrices that represent the individual MZIs.

For the sake of definiteness, in the following we use the so-called "triangular" mesh [6], which can be viewed as consisting of successive "diagonal line" self-configuring layers [3]. The conventions we use are shown in Fig. 2b of the main text. Each node corresponds to a 2×2 unitary operator between two adjacent nodes $(j, j+1)$ and written as:

$$T_{j,j+1}^{-1} = \begin{bmatrix} e^{-i\phi}\sin(\theta) & \cos(\theta) \\ e^{-i\phi}\cos(\theta) & -\sin(\theta) \end{bmatrix}.$$

This operator can be implemented as a combination of a phase shifter (value ϕ) with a tunable beamsplitter (ratio $\cos\theta$). The decomposition algorithm for this particular mesh of a matrix U (size $N \times N$) works as described in Ref. [6].

The decomposition algorithm multiplies the original matrix U on its right hand side by 2×2 unitary matrices:

$$T_{j,j+1} = \begin{bmatrix} e^{i\phi}\sin(\theta) & e^{i\phi}\cos(\theta) \\ \cos(\theta) & -\sin(\theta) \end{bmatrix}.$$

The resulting decomposition can be written as:

$$U = DT_{N-1,N}^{-1} \dots T_{1,2}^{-1} T_{2,3}^{-1} \dots T_{N-1,N}^{-1} \tag{S6}$$

$$= D\tau_{N-1} \dots \tau_1, \tag{S7}$$

where D is a diagonal matrix of phases and τ_i is the action of the i-th self-configuring layer of $T_{j,j+1}^{-1}$'s. Since the self-learning algorithm runs over average power outputs, the diagonal of phases

D is usually ignored. In the example of Fig. 2b of the main text for a 4×4 unitary,

$\tau_1 = T_{1,2}^{-1} T_{2,3}^{-1} T_{3,4}^{-1}$	corresponds to MZIs M_1
$\tau_2 = T_{2,3}^{-1} T_{3,4}^{-1}$	corresponds to MZIs M_2
$\tau_3 = T_{3,4}^{-1}$	corresponds to MZIs M_3

B. PCLA learning algorithm in triangular arrays

We now turn to the numerical implementation of the optimization algorithm described above for partially coherent light in triangular MZI arrays. The optimization algorithm is a sequential power optimization routine over ensemble (e.g., time) averaged outputs. Numerically, we simulate partially coherent light using random variables and the following reparametrization for the fluctuating fields x:

$$x = \mu + R\epsilon, \tag{S8}$$

where μ is the mean field amplitude of x, ϵ is a vector of independent and identically distributed (i.i.d.) (e.g., normal) random variables, and R is a square-root matrix of the desired coherency matrix: $RR^\dagger = \rho_{\text{in}}$. In our numerical experiments, this reparametrization is essential to automatically calculate gradients with respect to network parameters. Since calculating gradients with respect to stochastic variables can be challenging [9], the reparametrization trick allows us to separate stochasticity and functional variables (with respect to which gradients should be calculated) by only using random variables with fixed distributions (here, ϵ). For simplicity, and without loss of generality, in most numerical experiments we take $\mu = 0$. In all numerical implementations, we are using ADAM (gradient descent) [10] for optimization and TensorFlow's automatic differentiation methods, which are compatible with this reparametrization trick.

In Algorithm 1, we can either maximize or minimize the time-averaged power output, which corresponds to finding the eigenvalues of the coherency matrix in decreasing or increasing order, respectively. As discussed in Section S1, the result of this optimization routine is $U_{\text{PCLA}} = U^\dagger$, the Hermitian conjugate of the matrix that diagonalizes ρ_{in}, and the time-averaged power output of the network maps to the eigenvalues of ρ_{in}.

To characterize the quality of the solution of this optimization problem, we calculate the fidelity

Algorithm 1 PCLA learning algorithm for partially coherent light

Initialize network parameters (θ, ϕ) to random values.

Network input is fluctuating field (random variable) x described by coherency matrix ρ_{in}.

for ind_opt $\leftarrow 1$ **to** $N-1$ **do**

  Maximize (or minimize) time-averaged power at output index ind_opt using phases from matrices in $\tau_{\text{ind\_opt}}$. Time-averaged power is simulated by averaging N_S samples from the distribution as defined in Eq. S8: $\langle |(U_{\text{PCLA}} x)_k|^2 \rangle$.

  Update (θ, ϕ) in $\tau_{\text{ind\_opt}}$ accordingly.

end for

of the PCLA to the ground truth eigenmodes of the coherency matrix, normalized by the phases:

$$F = \langle |U_{\text{PCLA}} U|, \mathbb{1} \rangle_{\text{HS}}, \tag{S9}$$

where $\langle \cdot \rangle_{\text{HS}}$ is the Hilbert-Schmidt dot product, defined as:

$$\langle A, B \rangle_{\text{HS}} = \frac{\text{Tr}(A^\dagger B)}{\sqrt{\text{Tr}(A^\dagger A)\text{Tr}(B^\dagger B)}}. \tag{S10}$$

A fidelity of one means that $U_{\text{PCLA}} = U^\dagger$, up to a diagonal matrix of phases $e^{i\varphi_j}$. Results for a 10×10 coherency matrix ρ are shown in Fig. 2 of the main text.

S3. ANALYSIS OF QUANTUM OPTICAL MIXTURES WITH PCLA

We now generalize the algorithm from the previous section to processing of partially coherent quantum light, namely incoherent mixtures of single photons propagating in an integrated photonic network.

A. Background on density matrix

We consider a quantum optical state $|\psi\rangle$ which can be imparted an arbitrary unitary operator U_{PCLA} with an array of MZI: a single photon spatially delocalized over N ports $|i\rangle$. The output of the MZI network is $|\psi'\rangle = U_{\text{PCLA}} |\psi\rangle$ where U_{PCLA} is the classical unitary transformation imparted to light, as described in the previous sections. Let us consider an incoherent mixture of M such

single photon pure states, described by a density matrix $\rho_{\text{in}} = \sum_{j=1}^{M} p_j \left|\psi_j\right\rangle \left\langle\psi_j\right|$. The input-output relationship of the density matrix also holds in the case of an incoherent mixture [11].

Unlike the classical case considered in the previous section, stochasticity in the quantum system arises from incoherence of the mixture and projective measurements. Specifically, if the state to be measured is decomposed on the canonical basis as $\left|\psi_i\right\rangle = \sum_j A_{ij} \left|j\right\rangle$, we get the following expression for the density matrix:

$$\rho = \sum_{jk} \left(\sum_i A_{ji} A_{ki}^* \right) \left|j\right\rangle \left\langle k\right| . \tag{S11}$$

We can now derive the expression for our power maximization method, for instance on the output port labelled $\left|1\right\rangle$:

$$\begin{aligned} \langle P_1 \rangle &= \text{Tr}\left(P_1 \rho_{\text{out}}\right) && \text{(S12)} \\ &= \text{Tr}\left(P_1 U_{\text{PCLA}} \rho_{\text{out}} U_{\text{PCLA}}^{\dagger}\right), && \text{(S13)} \\ &= \langle U_{\text{PCLA}}^1 | \rho | U_{\text{PCLA}}^1 \rangle , && \text{(S14)} \end{aligned}$$

where U_{PCLA}^1 is the first row of U_{PCLA}. This yields the following relationship:

$$\max_{U_{\text{PCLA}}} \langle P_1 \rangle = \max_{U_{\text{PCLA}}^1} \langle P_1 \rangle = \lambda_1, \tag{S15}$$

where λ_1 is the largest eigenvalue of ρ_{in}. This process is therefore formally equivalent to classical incoherence modeled as field fluctuations, as in Section S2. By applying the same sequential power optimization, one can diagonalize (and analyze) the density matrix of such quantum optical systems in self-configuring networks.

B. PCLA algorithm for quantum optical system

The sequential power optimization algorithm is similar to that used of classical partially coherent light in Section S2, given the analogy between density and coherency matrices highlighted in the previous section. The main difference in its practical implementation is the way the stochasticity is parametrized, now on the output measurement side. We need to use another reparametrization trick to be able to calculate gradients with automatic differentiation, which we describe in the algorithm below (Algorithm 2).

This algorithm effectively works as follows:

Algorithm 2 Sampling routine for projective measurements from quantum mixtures

1: Given output wavefunction samples $|\psi_{\text{out}}\rangle$, output port $|i_0\rangle$, and number of samples N_S

2: **Pick wavefunction from mixture.** Pick N_S states from mixture $|\psi_{s(j)}\rangle$ according to categorical distribution parametrized by the mixture probabilities.

3: **Simulate projective measurements.** Calculate N_S corresponding projective measurement probabilities $|\langle\psi_{s(j)}|i_0\rangle|^2$

4: Draw sample $\epsilon \sim \mathcal{N}(0,1)$ from a normal distribution

5: Measured power on output port is $P_{i_0} = \epsilon\sigma + \mu$, where σ and μ are defined below.

1. First, draw N_S states $|\psi_{s(j)}\rangle$ from the incoherent mixture, according to the mixture's probabilities. Here, $s(j) \in \{0, \ldots, M-1\}$ for $j \in \{1, \ldots, N_S\}$
2. Second, draw a random number from a Bernouilli distribution with probability parameter $|\langle\psi_{s(j)}|i_0\rangle|^2$.
3. These two operations are averaged over N_S samples to yield the average power (in units of number of single-photon "clicks").

To perform automatic differentiation, we need to rely on a reparametrization trick based on a modified version of the central limit theorem. The mean number of clicks at port $|i_0\rangle$ is a sum of random variables:

$$X = \frac{1}{N_S}\sum_{j=1}^{N_S} X_j, \tag{S16}$$

with X_j drawn from a Bernouilli distribution (binary outcome 0 or 1) with parameter $|\langle\psi_{s(j)}|i_0\rangle|^2$. In the limit of many samples, this distribution can be approximated as:

$$X \sim \mu + \sigma\epsilon, \tag{S17}$$

where $\sigma = \frac{1}{N_S}\sqrt{\sum_j p_j(1-p_j)}$ and $\mu = \frac{1}{N_S}\sum_j p_j$, and ϵ is a random variable following a normal distribution. This generalized version of the central limit theorem (where the random variables in the sum have different means) is sometimes referred to as Lindeberg's theorem [12]. The Lyapunov condition, necessary to its application, is verified in our case. Another advantage of this method is that it "smoothens" the measurement function which, in principle, is the sum of many binary inputs (drawn from Bernouilli distributions), whose discrete nature would make calculating gradients problematic. Alternative methods could leverage recent work in automatic differentiation of stochastic binary variables [9].

C. Influence of losses

We now evaluate the influence of losses on the accuracy of the PCLA algorithm in reconstructing the density matrix of incoming light.

We assume that losses are uniform across channels and elements of the mixture, such that we can write

$$|\psi_i\rangle \to \sqrt{1-\alpha^2} U_{\mathrm{PCLA}} |\psi_i\rangle + \alpha^2 |\mathrm{vac}\rangle , \tag{S18}$$

where $1 > \alpha > 0$ is the uniform loss rate, and $|\mathrm{vac}\rangle$ is the vacuum state (resulting from the loss of a single photon propagating through the PCLA). This model can account for various loss mechanisms throughout the network (scattering, absorption, non-ideal detection efficiency, etc.) as long as they are uniform across modes. The resulting output density matrix is given by:

$$\rho_{\mathrm{out},\alpha} = (1-\alpha^2) U_{\mathrm{PCLA}} \rho_{\mathrm{in}} U_{\mathrm{PCLA}}^{\dagger} \tag{S19}$$

$$+ \sum_i p_i \alpha \sqrt{1-\alpha^2} \left(U_{\mathrm{PCLA}} |\psi\rangle \langle \mathrm{vac}| + U_{\mathrm{PCLA}}^{\dagger} |\mathrm{vac}\rangle \langle\psi| \right) + p_i \alpha^2 |\mathrm{vac}\rangle \langle \mathrm{vac}| . \tag{S20}$$

Only the first term contributes to the average power measured at output port k, therefore the PCLA will still find the corresponding eigenvector of the density matrix:

$$\mathrm{argmax}_{U_{\mathrm{PCLA}}} \left(\rho_{\mathrm{out},\alpha}\right)_{kk} = \mathrm{argmax}_{U_{\mathrm{PCLA}}} \left(\rho_{\mathrm{out,no\ loss}}\right)_{kk} . \tag{S21}$$

The corresponding eigenvalues are rescaled by the loss factor $1-\alpha^2$. In practical settings, the loss can be measured and used to estimate the eigenvalues of the density matrix.

S4. CONNECTION TO OTHER MODAL REPRESENTATIONS AND MEASUREMENT METHODS OF PARTIAL COHERENT LIGHT

In this section, we highlight connections between the modal representation of partial coherent light discovered by the PCLA and other decompositions found in the literature.

A. Coherence tomography

Partial polarization is a form of partial coherence of light. In polarization optics, it is known that a combination of projective measurements can reconstruct the full Stokes vector (or, equivalently,

the polarization coherency matrix), which represents a partially polarized light field [13]. Each projective measurement takes the form

$$P_i = \langle s|\Pi_i|s\rangle \,, \tag{S22}$$

where Π_i is a projector on a state of the Poincaré sphere $\Pi_i = |i\rangle \langle i|$ and $|s\rangle$ is the Stokes vector to be measured. By combining several P_i's taken on orthogonal polarization states, one can fully reconstruct $|s\rangle$.

This projective measurement method can be generalized to coupled degrees of freedom describing more complex forms of partial coherence [14] by using projectors acting jointly on the multiple degrees of freedom of interest (e.g., spatial and polarization). Since these methods are all based on projective measurements, they are intrinsically lossy and require further processing to reconstruct the full coherency matrix. Additionally, one distinctive advantage of our method is that it separates the field into its mutually incoherent components, which can then be used for further processing in "natural modes" basis [15, 16].

B. Karhunen-Loève expansions

Modal representations of partially coherent light field have been proposed by Wolf [15, 16]. These representations rely on Karhunen-Loève expansions of stochastic processes [17]. We first reproduce the main elements of Wolf's theory below before drawing a connection with the spectral decomposition of the coherency matrix.

We consider a stochastic process $x_i(t)$ where i is an index encoding a degree of freedom of the light field, for instance the spatial location at which the field is measured. Assuming that stochastic process is stationary, we define the autocorrelation of the field:

$$\Gamma_{ij}(\tau) = \langle x_i(t)x_j^*(t+\tau)\rangle_t, \tag{S23}$$

which is an ensemble average over different temporal realizations of the stochastic process.

In the numerical examples of our work, we consider white noise processes, which means that the cross-spectral density – the Fourier transform of the field autocorrelation – is independent of frequency and takes the form:

$$W_{ij} = \frac{1}{2\pi}\langle x_i x_j^*\rangle, \tag{S24}$$

where the x_i's are i.i.d. random variables. More generally, Wolf showed that there exists a set of monochromatic oscillations that provide a representation of the cross-spectral density as an ensemble average in frequency domain [15]. This definition of the cross-spectral density is equivalent to the coherency matrix of the light field used in this paper.

Using Mercer's theorem, the cross-spectral density may be expanded as [15]:

$$W_{ij} = \sum_n \lambda_n U_{in} U_{jn}^*, \tag{S25}$$

where U is an orthogonal basis and $\lambda_n \geq 0$. This is formally equivalent to the spectral decomposition of the coherency matrix we use in this paper:

$$(\rho_{\text{in}})_{ij} = \left(UDU^\dagger\right)_{ij} = \sum_n \lambda_n U_{in} U_{jn}^*. \tag{S26}$$

S5. DISCUSSION OF SELF-CONFIGURING ALGORITHM FOR A LAYER WITH PARTIALLY COHERENT LIGHT

With a fully coherent input field, we know that with sets of MZIs or equivalent controllable beamsplitters, we can proceed sequentially, minimizing the power at the "drop" port of each one (actually to zero) to maximize the overall output from a set or layer of interferometers in a self-configuring topology [3–5]. In the case of partially coherent fields, though we can still establish the eigenvectors of the coherency matrix by maximizing the output power in such layers, we cannot guarantee to do this by minimizing the power at each such drop port. We can prove this by counter example.

Consider the three beamsplitters as in Fig. S2. These beamsplitters can be MZIs, but for simplicity of discussion we show them as cube beamsplitters. We imagine we have two different source beams that are fully mutually incoherent. One such beam from a source A shines with a power $P_1 = 2$ (in arbitrary units) on the input to "beamsplitter" BS1. A second source, B, which is fully mutually incoherent with source A, generates two beams that are fully coherent with one another – one with power $P_2 = 1$ on BS2, and a second with power $P_3 = 3$ on BS3.

Note, first, that, if we have two fully incoherent beams incident on the two input ports of a beam splitter, such as the P_1 on the left of BS1 and the P_2 on the top of BS1, the choice of reflectivity R_2 that minimizes the "dropped" power P_{T2} is simply to choose to route all of the larger power

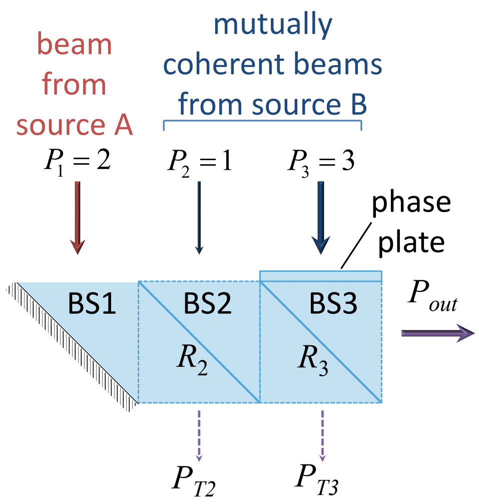

FIG. S2: **Conceptual apparatus for counter-example proof. a.** A schematic conceptual drawing of three beamsplitters, one after another in a row, for use in our "thought experiment" proof by counter example. The first "beamsplitter" BS1 is actually just a fully reflecting mirror at 45°, but beamsplitters BS2 and BS3 can have chosen reflectivities R_2 and R_3 respectively. We also allow for a chosen phase delay on the input to BS3, given by a phase plate of a chosen thickness. A beam of power $P_1 = 2$ units from a source A shines on BS1. Source B, which is fully mutually incoherent with source A, generates two mutually coherent beams, one with power $P_2 = 1$ on BS1, and a second with power $P_3 = 3$ on BS2.

to the right. In this case, since $P_1 > P_2$, the choice of R_2 that minimizes P_{T2} is to make $R_2 = 0$. This transmits $P_1 = 2$ to the right into BS3, and transmits $P_2 = 1$ to become P_{T2}. Now BS3 also has two mutually incoherent inputs, with $P_3 = 3$ being the larger one, so now the choice that minimizes P_{T3} is to make $R_3 = 1$. So $P_{out} = P_3 = 3$ (and, incidentally, all of P_1 and P_2 have been discarded). However, this is not the choice of reflectivities that maximizes P_{out}. We can show this by finding another solution with $P_{out} > 3$. Specifically, choose $R_2 = 1$, reflecting all of P_2 into BS3. Now, because the two beams P_2 and P_3 are mutually coherent, there is a choice of R_3 and the phase plate thickness that allows us to combine both these powers fully, therefore giving $P_{out} = 4$, which is greater than what we obtain by successive minimization of P_{T2} and P_{T3}, which was $P_{out} = 3$.

Hence, adjusting reflectivities (and possibly phase plate thicknesses) to successively minimize the "drop-port" powers P_{T2} and P_{T3} does not guarantee the maximum possibly value of P_{out}. This completes our proof by counter example. (Note, incidentally, that we are not computing the setting that overall gives the largest output power, though we could readily do so. It is sufficient for this

proof that we have found some settings that give $P_{out} > 3$.)

Hence, the algorithm that maximizes the power at the output will in general have to be a global one within this "layer" of the system. It is still true, however, that we can optimize each successive layer separately and sequentially.

S6. EXPERIMENTAL CONSIDERATIONS

In this section, we further elaborate on experimental realizations of PCLAs and their potential applications.

A. Experimental proposal

First, we include a concrete example of partially coherent light sources that could be analyzed with our device. The special case we chose is that of a set of mutually incoherent sources placed in the far field of the PCLA. Light propagation between the source plane and the input plane of the chip results in a partially coherent light field at the input, which can be processed by the PCLA using the method we propose in our work (see Fig. S3).

We also include in Fig. S4 a schematic of an experimental setup to analyze partially coherent light with the PCLA, for instance that generated by an array of incoherent LEDs in the far field, as in Fig. S3. Upon free space propagation, the light field acquires spatial partial coherence, and is then coupled into the PCLA chip with a lenslet array and a set of grating couplers. With this simple proof-of-concept experiment, one could already tune the input coherency matrix and see how the PCLA learns its modal representation for various distances between the LED array and the input plane of the chip.

B. Influence of additional sources of noise on fidelity

We now analyze the influence of additional sources of noise (that are not due to fluctuations of the input fields) on the fidelity of the learned matrix by the PCLA, shown in Fig. S5. We consider the case of detection noise of a given signal-to-noise ratio (SNR) for various random matrices of

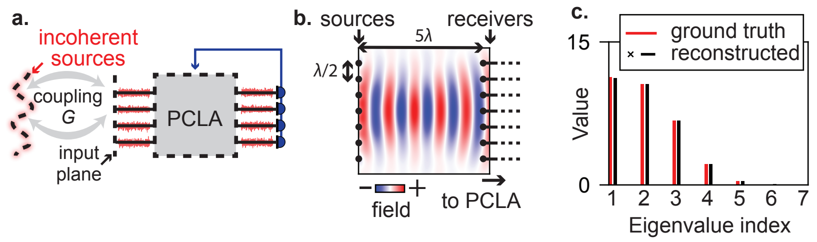

FIG. S3: **Concrete example of PCLA application.** **a.** Envisioned experimental configuration: a set of mutually incoherent sources are placed in the far field of the PCLA and coupled to its input plane via coupling operator G. **b.** Example geometry with 7 sources and receivers. The field distribution of the mode with the largest singular value is shown (multiplied by z where z is the distance to the source plane). **c.** Corresponding ground truth eigenvalues of the input coherency matrix and reconstructed values from PCLA.

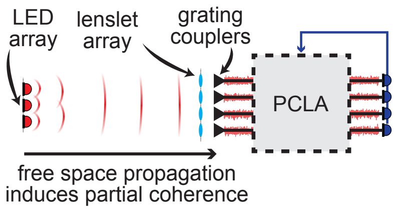

FIG. S4: **Experimental setup for PCLA.** Schematic of the experimental setup for a proof-of-concept experiment: an array of incoherent LEDs emits a light field that displays spatial partial coherence after a certain distance (due to the van Cittert-Zernike theorem). That spatially partially coherent light field is coupled into the chip with a set of lenslets and grating couplers. The PCLA analyzes the in-coupled light according to the algorithm presented in this manuscript.

size $N = 5$ and condition numbers. We also show that with relatively small numbers of iterations ($\text{n\_epochs} = 20$) and SNR on the order of 1, the learned fidelities are > 0.9. This illustrates the robustness of the PCLA to additional sources of noise.

References

[1] J. W. Goodman, *Statistical optics*. Hoboken: John Wiley & Sons, 2015.

[2] H. W. Zhang, C. W. Hsu, and O. D. Miller, “Scattering concentration bounds: brightness theorems for waves,” *Optica*, vol. 6, no. 10, pp. 1321–1327, 2019.

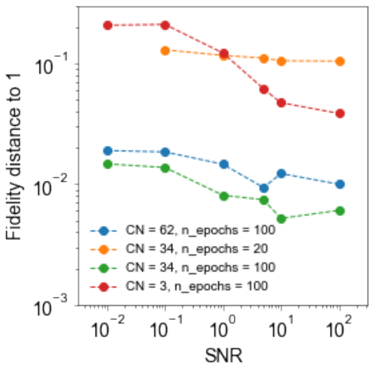

FIG. S5: **Influence of signal-to-noise ratio (SNR) on algorithm fidelity.** The fidelity distance to 1 (defined as 1 - F, where F is the fidelity) is plotted for random coherency matrices of various condition numbers (CN) and running the learning algorithm for different numbers of iterations ($\mathrm{n\_epochs}$). All simulations are run for an input field distributed over $N = 5$ spatial ports.

[3] D. A. B. Miller, "Self-configuring universal linear optical component," *Photonics Research*, vol. 1, no. 1, pp. 1–15, 2013.

[4] D. A. B. Miller, "Analyzing and generating multimode optical fields using self-configuring networks," *Optica*, vol. 7, pp. 794–801, 2020.

[5] D. A. B. Miller, "Self-aligning universal beam coupler," *Optics express*, vol. 21, no. 5, pp. 6360–6370, 2013.

[6] M. Reck *et al.*, "Experimental realization of any discrete unitary operator," *Physical review letters*, vol. 73, no. 1, pp. 58–61, 1994.

[7] A. Annoni, E. Guglielmi, M. Carminati, G. Ferrari, M. Sampietro, D. A. Miller, A. Melloni, and F. Morichetti, "Unscrambling light—automatically undoing strong mixing between modes," *Light: Science & Applications*, vol. 6, no. 12, pp. e17110–e17110, 2017.

[8] D. A. B. Miller, "An introduction to functional analysis for science and engineering," *arXiv preprint arXiv:1904.02539*, 2019.

[9] G. Arya, M. Schauer, F. Schäfer, and C. Rackauckas, "Automatic differentiation of programs with discrete randomness," *Advances in Neural Information Processing Systems*, vol. 35, pp. 10435–10447, 2022.

[10] D. P. Kingma and J. Ba, "Adam: A method for stochastic optimization," *Proceedings of the 3rd International Conference on Learning Representations. San Diego: ICLR*, 2015.

[11] M. A. Nielsen and I. L. Chuang, *Quantum computation and quantum information*. Cambridge: Cambridge university press, 2010.

[12] R. B. Ash and C. A. Doléans-Dade, *Probability and measure theory*. Academic press, 2000.

[13] J. N. Damask, *Polarization optics in telecommunications*, vol. 101. Springer Science & Business Media, 2004.

[14] K. H. Kagalwala *et al.*, "Optical coherency matrix tomography," *Scientific Reports*, vol. 5, no. 1, p. 15333, 2015.

[15] E. Wolf, "New theory of partial coherence in the space–frequency domain. part i: spectra and cross spectra of steady-state sources," *Journal of the Optical Society of America*, vol. 72, no. 3, pp. 343–351, 1982.

[16] E. Wolf, "New theory of partial coherence in the space–frequency domain. part ii: Steady-state fields and higher-order correlations," *Journal of the Optical Society of America A*, vol. 3, no. 1, pp. 76–85, 1986.

[17] R. G. Ghanem and P. D. Spanos, *Stochastic finite elements: a spectral approach*. Courier Corporation, 2003.
